# In vivo detection of programmed cell death during mouse heart development

**DOI:** 10.1038/s41418-019-0426-2

**Published:** 2019-09-30

**Authors:** Kristel Martínez-Lagunas, Yoshifumi Yamaguchi, Cora Becker, Caroline Geisen, Marco C. DeRuiter, Masayuki Miura, Bernd K. Fleischmann, Michael Hesse

**Affiliations:** 1 Institute of Physiology I, Life and Brain Center, Medical Faculty, University of Bonn, Bonn, Germany; 2 Department of Genetics, Graduate School of Pharmaceutical Sciences, The University of Tokyo, Tokyo, Japan; 3 Hibernation Metabolism, Physiology, and Development Group, Environmental Biology Division, Institute of Low Temperature Science, Hokkaido University, Kita 19, Nishi 8, Kita-ku, Sapporo, Hokkaido, 060-0819 Japan; 4 Department of Anatomy and Embryology, Leiden University Medical Center, Leiden, The Netherlands

**Keywords:** Cell biology, Development

## Abstract

Despite the great progress on the cell biology of programmed cell death (PCD), its incidence and exact time course during embryonic and particular heart development are still unclear. This is also due to the lack of models enabling to directly identify and monitor PCD cells at different time points in vivo. Herein we report generation of transgenic murine embryonic stem cell and mouse models expressing secreted Annexin V-YFP under control of the CAG promoter. This enables to visualize and quantify PCD in vitro and in vivo during embryonic development. At early embryonic stages we found Annexin V-YFP^+^ fluorescent cells in known areas of PCD, such as the otic ring and at the site of neural tube closing, underscoring its specificity for detection of PCD. We have focused our detailed analysis primarily on PCD in the embryonic heart for a better understanding of its role during development. Our findings reveal that PCD peaks at early stages of cardiogenesis (E9.5–E13.5) and strongly decreases thereafter. Moreover, the PCD cells in the heart are predominantly cardiomyocytes, and an unexpected area of prominent cardiac PCD are the ventricular trabeculae (E9.5–E14.5). Thus, the sA5-YFP mouse line provides novel insight into the incidence and relevance of cardiac PCD during embryonic development ex- and in vivo.

## Introduction

Programmed cell death (PCD) is an important event during embryonic development enabling the correct formation of different organs [1–3]. Currently, there exist at least 13 different modalities of PCD [4].

To date, the detection and quantification of PCD^+^ cells have been mostly assessed in histological tissue samples using methods such as terminal dUTP nick end labeling (TUNEL) to detect DNA fragmentation and/or staining with antibodies against cleaved caspases (cleaved caspases 3, 7, 8, or 9) or Annexin V conjugates making the assessment of PCD tedious, time consuming, and expensive [5]. In addition, most of these techniques have limitations to accurately and specifically detect PCD^+^ cells [5] as they label PCD^+^ cells at a single endpoint of the PCD cascade. Given that the progression of PCD events occurs within minutes [6, 7], current methods do not provide enough information regarding the origin, extent, and kinetics of the PCD cascade limiting the understanding of its pathophysiological role. Thus, a more specific in vivo detection method would be warranted to overcome these limitations to detect PCD^+^ cells in vivo.

The Annexin V-based assay is one of the most sensitive techniques to detect exposure of phosphatidylserine (PS) residues in the outer membrane of cells undergoing PCD in vitro, ex vivo, and in vivo [8, 9]. This method detects cells in various phases of the PCD cascade ranging from the early phase prior to morphological nuclear changes until the late phase [10]. However, this technique faces limitations, when used in vivo, therefore strongly limiting this tool for its in vivo usage [5]. To overcome these limitations and to assess PCD in vivo we have generated an Annexin V-based encoded reporter tool used to visualize and quantify PCD in mouse cells and embryonic tissues.

As a proof of concept, we have focused in the present manuscript on the heart, because of the difficulties of identifying PCD events in this organ and its importance for development and disease processes. Several studies suggest that PCD is important for the shaping of the heart [11–13], as knockout mouse models for genes mediating PCD develop heart defects. Deletion of caspases 3, 7, or 8 leads to aberrant ventricular chamber formation including thin and disorganized trabeculae and hemorrhage, causing early embryonic lethality around E11 [14, 15]. Studies performed in mice have located preferential sites of PCD in the developing heart, namely the outflow tract (OFT), endocardial cushions, ventricular wall, intraventricular septum, and the atria [12]. It has been suggested that both enhanced or reduced rates of PCD are related to congenital and ischemic heart disease [16–19]. Nonetheless, cell type, number, and location of these PCD cells are controversial [20, 21] illustrating that a big challenge is its accurate detection and quantification in vivo [22].

Herein we report the establishment of transgenic embryonic stem (ES) cells and a mouse line that stably express a secreted Annexin V (sA5) protein fused to yellow fluorescent protein (YFP) under control of the ubiquitous β-actin promoter with a CMV enhancer element (CAG). We have validated the functionality of the CAG-sA5-YFP system as a PCD marker in vitro and in vivo, and used it to provide detailed insight into extent and localization of PCD in the developing mouse heart.

## Methods

### Generation of the CAG-sA5-YFP expression cassette

We generated the CAG-sA5-YFP expression construct (6995 bp) by standard cloning techniques, using the pBH-UAS-secA5-YFP vector [9] (kindly provided by Randall T. Peterson, University of Utah, UT, USA) and the CAG-Neo3-eGFP (Clontech Laboratories, Inc.) vector.

### Generation of CAG-sA5-YFP transgenic mouse ES cells (mESCs)

G4 hybrid ESCs [23] were tested negative for mycoplasma contamination and cultured in Knockout Dulbecco’s modified Eagle’s medium (Invitrogen), high-glucose, supplemented with 15% v/v fetal calf serum (Promega), 0.1 mM nonessential amino acids (Invitrogen), 2 mg/ml l-Glutamine (Invitrogen), 50 μg/ml each penicillin and streptomycin (Invitrogen), 0.1 mM β-mercaptoethanol (Sigma), and 500 U/ml leucemia inhibitory factor (Chemicon). The ESCs were kept on irradiated murine fibroblasts derived from neomycin resistant mice.

mESCs were transfected with the CAG-sA5-YFP plasmid by electroporation. After neomycin (Geneticin Sulfate, Gibco/Life Technologies) selection, resistant clones were isolated under fluorescence light to detect YFP expression. Clones with stable YFP expression were analyzed by fluorescence microscopy and qPCR. For the qPCR analysis, we used RNA isolated from wild-type G4-mESCs (provided by Nagy Lab, Toronto, Canada) [23] as negative control and RNA from an mESC line stably expressing EYFP (kindly donated by Tobias Bruegmann, Institute of Physiology I, University of Bonn, Germany) as positive control. The newly generated mESCs were karyotyped and further cultured for generation of a transgenic mouse line. Offspring were viable and did not show an overt phenotype.

### PCD induction in sA5-YFP mESCs

PCD was induced by treating mESCs with 40 µm of hydroxytamoxifen (4-OHT) [24] or 1% DMSO [25].

### Live-cell imaging of PCD^+^ cells in mESCs

Transgenic and non-transgenic mESCs were grown on gelatin-coated 24-well plates (In Vitro Scientific) under culture conditions described above. At 60% confluency PCD was induced by 4-OH treatment and non-transgenic cells were treated with 1 µM Annexin V-FITC (Calbiochem/Millipore) for 30 min at 37 °C before 4-OHT addition. Afterward, images were taken every 5 min for 24 h with the fluorescence microscope Observer Z1 (Carl Zeiss), the Colibri illumination system (blue and white LED), and the 20× air objective, the transmission light and GFP filters.

### Generation of CAG-sA5-YFP transgenic mice and determination of transgene integration number

The CD-1-Tg(CAG-sA5-YFP)^KML^ mouse line was generated from a CAG-sA5-YFP mESC line by complementation with diploid embryos from wild-type CD-1 mice as previously described [23]. The number of integrations of the transgene into the genome was determined as one by qPCR with a probe detecting YFP/GFP and normalized to a transgenic mouse line with one copy of a GFP-anillin expression cassette [26]. Transgenic CAG-sA5-YFP mice were crossed with mice from the CD-1 outbred strain to maximize the number of embryos per gestation.

### Animal procedures

All animal experiments were performed in accordance with the Guide for the Care and Use of Laboratory Animals published by the National Institutes of Health (8th edition, revised 2011) and were approved by the local ethics review board (Landesamt für Natur, Umwelt und Verbraucherschutz Nordrhein-Westfalen, Germany, #84-02.04.2012.A146).

### Isolation and fixation of mouse embryos and embryonic hearts

Mouse embryos at different embryonic stages (E9.5–P2) were taken out from pregnant mice at the respective day. The day of embryonic development was determined by counting the day of the vaginal plug as day 0.5. The pregnant mice were killed by cervical dislocation and the embryos were dissected and then placed in ice-cold PBS, 2.5 mM CaCl_2_ (Invitrogen/Life Technologies). The placenta and in some cases also the yolk sac were removed. Embryos were scanned under a fluorescent macroscope to distinguish transgenic from wild-type embryos. Immediately after embryos were fixed in 4% paraformaldehyde (PFA), 2.5 mM CaCl2 in PBS for 2–4 h, they were washed and incubated in 20% sucrose, 2.5 mM CaCl_2_ for 24 h.

For the isolation of embryonic hearts (E11.5–P2), embryos were decapitated, the thorax was opened, and the heart was taken out with small forceps. All connective tissue was removed and hearts were then fixed in the same way as the isolated embryos.

### Histology and immunofluorescence staining

Prior to immunofluorescence staining, all cells were fixed with 4% PFA in PBS with 2.5 mM CaCl_2_. Cryopreserved embryonic tissues were sectioned with a cryotome into 10 µm thick slices.

Fixated cells were permeabilized at RT with 0.2% (v/v) Triton X-100 in DPBS for 10 min. Cells and tissue slices were stained for the following antibodies (in 0.2% Triton X in PBS, supplemented with 5% donkey serum; 2 h at room temperature): vimentin (1:800, AB5733, Chemicon), α-actinin (1:400, A7811, Sigma-Aldrich), MF20-eFluor 660 (1:200, 50-6503-80, eBioscience), α-smooth muscle actin (1:400, A5228, Sigma-Aldrich), cCasp3-,7-,8 (1:200, 9661L, 9491S, 8592S, Cell Signaling Technology), anti-eGFP (1:50, sc-5385, Santa Cruz Biotechnology), PECAM (1:800, 553370, Becton Dickinson), CD45 (1:50, 05-1416, Chemicon), and human Annexin V (1:50, ab54775, Abcam). For the eGFP, Annexin V, and cCasp3-,7-,8 stainings a short antigen-retrieval with 10 mM sodium citrate, pH 6.0, was performed in a KOS Microwave for 15 min at 93 °C (Milestone).

Primary antibodies were visualized by secondary antibodies conjugated to Cy3 or Cy5 (1:400, 705-165-147, 705-175-147, Jackson ImmunoResearch) diluted in Hoechst 33342 (nuclei staining) at room temperature for 1 h. Pictures were taken with an inverted fluorescence microscope (Axiovert 200; Carl Zeiss MicroImaging, Inc.) equipped with a slider module (ApoTome; Carl Zeiss MicroImaging, Inc.).

For the detection of late PCD^+^ cells, the In Situ Cell Death, fluorescein detection kit (Roche) was used. Briefly, sample sections were washed twice with DPBS and treated with TUNEL reaction mix followed by incubation for 1 h at 37 °C. Finally, slides were quickly washed with DPBS and mounted. The detection of PCD^+^ cells was analyzed under a fluorescence microscope (Observer Z1, Carl Zeiss) using FITC and Cy3 filter sets.

### Live imaging of A5-YFP embryos and hearts at E8.75-9.5

Pregnant mice were killed by cervical dislocation at day 8.75–9.5 post coitum. The uterus was dissected and immersed in 37 °C warm Opti-MEM (Gibco/Life Technologies) culture medium and embryos removed from the uterus and yolk sac under a stereomicroscope (Microscope S8AP0, Leica). Embryos were scanned under a fluorescent macroscope to select for sA5-YFP expression and heart beating. Approximately 500 µl of 2% low-melting agarose (Carl Roth) was poured into an imaging chamber with a glass bottom. Holes were dug into the agarose before embryo dissection. Selected embryos were placed in the agarose holes and the imaging chamber was filled with IMDM imaging medium. Afterward, the chamber was positioned into an inverted confocal microscope (Laser Scanning Microscope Eclipse Ti, Nikon instruments) live imaging system and incubated at 37 °C and 5% CO_2_. Whole body or the heart of transgenic sA5-FYP embryos was imaged for 15–20 h. Pictures were taken every 5–10 min using a 10× air objective. YFP was excited by a 515 ± 30 nm laser (8–15% power) and a 595 ± 50 nm laser (8–15% power) was used to excite autofluorescence. The transmission light source was simultaneously used. Z-stack slices depth was defined between 4–6 µm, depending on the experiment.

### Live imaging of neural tube closure in E8.0 sA5-YFP embryos

All media and dishes were pre-warmed to 37 °C before the embryos were dissected. Three hundred microliters of 2% low-melting agarose were poured into a 35 mm glass-bottom dish, and depression for embryos were formed at least 1 h before filling the agarose-mounted dish with culture medium. Pregnant mice were killed by cervical dislocation at day 8 post coitum. The uterus was dissected and immersed in pre-warmed (37 °C) HBSS (Gibco, Thermo Fisher Scientific) and embryos removed from the uterus and their yolk sacs under a stereomicroscope. Embryos were scanned under a fluorescent stereomicroscope (MZ16F, Leica) to select for sA5-YFP expression. Embryos were transferred into the depression on the agarose-mounted glass-bottom dish filled with culture medium (OPTI-MEM) (Gibco, Thermo Fisher Scientific) containing penicillin/streptomycin (Gibco/Life Technologies) and 50%-rat IC-serum (Charles River Laboratories), and positioned under an inverted confocal microscope (SP5, Leica). The embryos were imaged at 37 °C and 5% CO_2_ for 10 h. Pictures were taken every 3 or 6 min using a 10× objective lens with 2× zoom. YFP was excited by a 514 nm argon laser (20% power) with 458/514 nm dichroic filter and the pinhole adjusted to 2 AU. The transmission light source was used simultaneously. Twenty z-stack slices of 10 μm were taken. Videos represent a maximum projection of all z-stacks.

### Caspase inhibition by zVAD-fmk

For inhibition of caspase activity in mESCs, 3 × 10^4^ transgenic mESCs per well were seeded in 24-well glass-bottom plates. When cells were at ~70% confluency, 50 µM of zVAD-fmk (Abcam) was added 2 h before cell death induction with 1% DMSO, cells were fixed after 24 h.

### Quantification and statistical analysis

The fluorescence measurements in mESCs were performed using Image J, version 1.37A software. Briefly, transgenic CAG-sA5-YFP mESCs either induced or non-induced with 4-OHT for 24 h, were first fixed with 4% PFA, 2.5 mM CaCl_2_ for 20 min at RT, stained for cleaved caspase 3 (cCasp3), and imaged using a fluorescence microscope (Observer Z1, ZEN 2 Blue Edition Software). Pictures of at least 4–6 areas of three different wells of transgenic induced, transgenic non-induced and wild-type induced cells were taken. Mean fluorescence intensity of each area in the green channel was quantified using the tool “integrated density measurements” from Image J. Sample’s total fluorescence was calculated by deducting sample’s fluorescence from background’s fluorescence. For statistical analysis, two different experiments were performed.

For the estimation of PCD rates during heart development, we quantified all different types of particles marked with sA5-YFP, cCasp3, or both with and without nucleus in CAG-sA5-YFP hearts from E9.5 to P2 in at least three hearts per developmental stage. The same analysis was performed for embryonic hearts after TUNEL staining. All numbers were normalized to the total number of nuclei. We defined a “PCD cell” as the one containing a nucleus and being positive for either cCasp3, sA5-YFP, or both. Similarly, a PCD body was defined as an entity not containing a nucleus and being positive for either cCasp3 or sA5-YFP or both.

Finally, for the quantification of cell types undergoing PCD we counted the sA5-YFP^+^ PCD^+^ cells and their fragmented cell bodies (PCD bodies) that colocalize with each cell type marker in at least three CAG-sA5-YFP hearts at E13.5. PCD bodies were defined as being positive for cCasp3 and/or sA5-YFP and not containing DNA. Total nuclei in ESCs and heart sections were quantified by the Axiovision Software, version 4.8.2.0. Data are depicted as mean ± SEM. Normal distribution of data was determined by D’Agostino and Pearson omnibus normality test. To test for statistical significance a one-way analysis of variance (ANOVA) with Tukey post hoc test or a Kruskal–Wallis statistic with Dunn’s multiple comparison post hoc test was used. P-values < 0.05 were considered as statistically significant (*) if P < 0.05, as very significant (**) if P < 0.01, and as extremely significant (***) if P < 0.001.

## Results

### Generation of the CAG-sA5-YFP PCD detection system

To assess PCD in vivo, and specifically, in the developing mouse heart, we have generated an Annexin V-based reporter tool to visualize and quantify PCD in mouse cells and tissues. The reporter consists of a CAG-sA5-YFP expression cassette [9] (Supplementary Fig. 1a), in which the ubiquitous CAG promoter drives the expression of a fusion protein consisting of a secretion signal peptide(s), the human Annexin V (A5) and YFP. When cells undergo PCD, the sA5-YFP protein is expected to bind to exposed PS residues in the presence of Ca^2+^-ions, resulting in bright fluorescent signals (Supplementary Fig. 1b).

### sA5-YFP reporter system labels PCD cells in mESCs

**Fig. 1:**
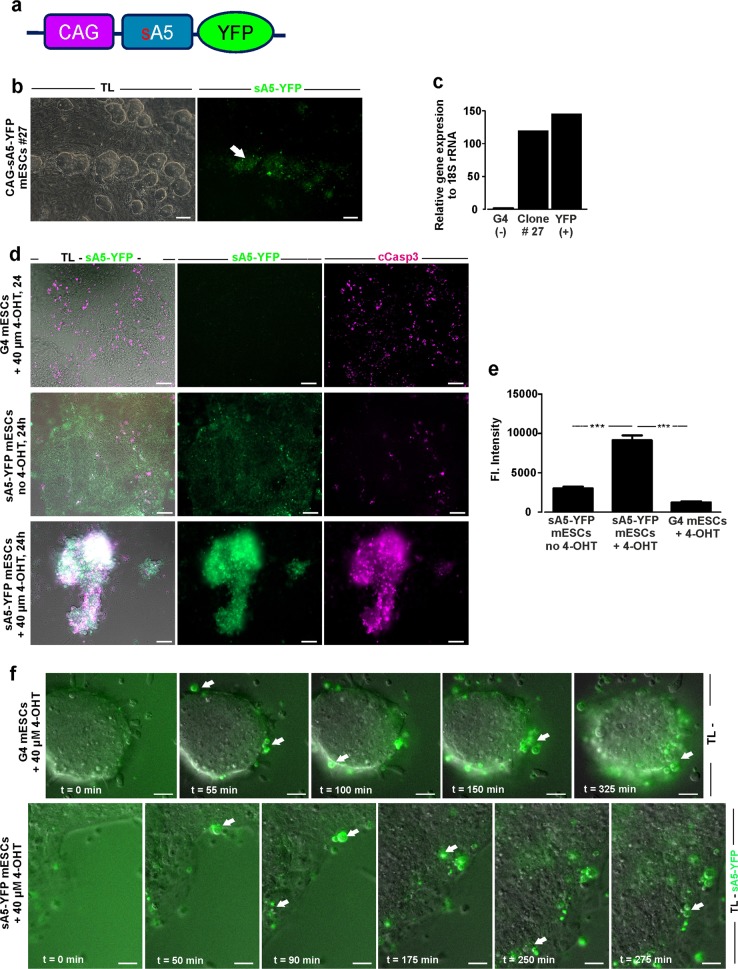
PCD detection in WT and CAG-sA5-YFP transgenic mESCs. **a** Scheme of the CAG-sA5-YFP construct to visualize PCD cells in mice. CAG chicken β-actin promoter with CMV enhancer, s secretion signal, A5 Annexin V, and YFP yellow fluorescent protein. **b** Transgenic mESCs stably expressing sA5-YFP (transmitted light and fluorescence pictures, left and right panels, respectively). Note weak intracellular background fluorescence of YFP overlapping with clusters of mESCs (white arrow); bright spots mark PCD cells. **c** Gene expression levels of sA5-YFP measured in a stable expressing mESC clone compared with wild-type G4 and mESC clones expressing YFP. **d** Representative fluorescent images revealing the accumulation of sA5-YFP in the membrane of PCD cells and colocalization of YFP^+^ (green) and cCasp3^+^ (magenta) signals after PCD induction by 40 µM 4-OHT (*n* = 3). Upper panel displays WT mESCs 24 h after PCD induction by 40 µM 4-OHT with cCasp3 signals, middle panel sA5-YFP transgenic mESCs without PCD induction, and lower panel sA5-YFP transgenic mESCs 24 h after PCD induction. **e** Quantification of sA5-YFP fluorescence intensities (FI) from **c**: FI of YFP significantly increased 24 h upon PCD induction by 4-OHT addition (*n* = 2). **f** Time-lapse microscopy (one frame every 5 min) monitoring onset and fate of PCD cells in WT mESCs preincubated prior to the experiment with 1 µM Annexin V-FITC (upper row) for 30 min and transgenic sA5-YFP mESCs (lower row) upon PCD induction (4-OHT at 40 µM for 24 h, *n* = 3). Examples of PCD cells labeled by Annexin V-FITC or sA5-YFP are depicted by arrows. G4 (−) G4 mESC line, YFP (+) mESC line expressing EYFP, 4-OHT 4-hydroxytamoxifen, TL transmission light, sA5-YFP secreted human Annexin V-yellow fluorescent protein, and cCasp3 cleaved caspase 3. Data in **e** are shown as mean ± s.e.m., ***P < 0.001 (Kruskal–Wallis statistic with Dunn’s multiple comparison post hoc test), *n* = 2. Bars are 50 µm (**b**, **c**) and 20 µm (**f**)

To explore the functionality of the sA5-YFP protein as a marker of PCD, we first generated transgenic G4-mESCs stably expressing the fusion protein by random integration of the expression cassette. Fluorescence microscopy and qPCR analysis revealed stable sA5-YFP expression in transgenic mESC clones (Fig. 1b, c and Supplementary Fig. 2a). As reported for a transgenic zebrafish overexpressing sA5-YFP [9], we could also observe background fluorescence which was caused by unbound sA5-YFP.

Next, we tested, whether we could detect bright sA5-YFP^+^ signals in our sA5-YFP mESCs upon induction of PCD. For induction of PCD in G4-mESCs we tested different substances and found 40 µM hydroxytamoxifen (4-OHT) and 1% DMSO to be most efficient (data not shown). Within 24 h after addition of 4-OHT G4 and sA5-YFP-mESCs became roundish, detached, and displayed morphological features typical for PCD (Fig. 1d, first and third rows), while non-induced sA5-YFP-mESCs colonies did not display morphological changes (Fig. 1d, second row). Importantly, bright YFP^+^-fluorescence signals could be observed in 4-OHT exposed transgenic mESCs (Fig. 1d, third row). In contrast, the YFP fluorescence intensity did neither increase in control sA5-YFP-mESCs without nor in G4-mESCs with 4-OHT addition (Fig. 1d, second and first rows, respectively) proving that the bright sA5-YFP^+^ signals could be exclusively detected in cells exposed to PCD stimulus.

Next, we measured changes in YFP fluorescence intensity and determined an ~3-fold increase upon exposure of the cells to 4-OHT compared with controls (Fig. 1e, *n* = 2). Furthermore, we stained against cCasp3 and found colocalization of sA5-YFP^+^ with cCasp3^+^ signals in sA5-YFP mESCs 24 h after induction of PCD (Fig. 1d, third row), but not in controls (Fig. 1d, second row). Given that cCasp3 is a key marker of early–intermediate caspase-dependent PCD [27, 28], our data suggest that sA5-YFP labels PCD cells in mESCs. To assess the utility of our novel genetic system in more detail, we directly compared fluorescence and detection patterns with that of the established Annexin V-FITC probe. We performed time-lapse microscopy and monitored the morphological changes occurring in PCD cells over time upon addition of 4-OHT (*n* = 3). Control G4-mESCs were exposed to an Annexin V-FITC probe prior to 4-OHT addition. Time-lapse microscopy illustrated sA5-YFP^+^ cells shedding from mESCs colonies with a clear accumulation of the sA5-YFP protein in their outer membranes within 30–50 min after PCD induction (Fig. 1f, white arrows in the lower row and Video 1). These bright sA5-YFP^+^ cells started to form fragmented cell bodies (PCD bodies) ~20–40 min later. We defined PCD bodies as being positive for cCasp3 and/or sA5-YFP and not containing DNA. After ~2.5 h of PCD induction, the PCD bodies were found to further fragment into smaller vesicles and to remain there for the next 3 h. These dynamic morphological changes of the cells were very similar to those observed with the Annexin V-FITC probe (Fig. 1f, arrows in the upper row, Video 2). The results underscored that our genetic sA5-YFP reporter is a highly suitable tool for live imaging of PCD cells.

### sA5-YFP detects also caspase-independent cell death in vitro

Necroptosis has been described as caspase-independent PCD, during which PS residues are externalized prior to the loss of cell integrity [29, 30]. Therefore, we tested if the sA5-YFP system could detect cells undergoing caspase-independent PCD. We cultured transgenic sA5-YFP mESCs in the presence or absence of zVAD-fmk (50 µM, 2 h), a widely used pan-caspase inhibitor [31, 32], induced PCD by addition of 1% DMSO and fixed and stained the cells for cCasp3. In most cells (*n* = 2 experiments) treated with zVAD-fmk cCasp3 was inhibited, as we could not observe anti-cCasp3 staining. However, sA5-YFP^+^ cells were still detectable after treatment (Supplementary Fig. 2b), showed a condensed nucleus and had a discontinuous line-like sA5-YFP^+^ signal surrounding their membrane (Supplementary Fig. 2b, white arrowheads and close-up), an indication for membrane permeabilization, which is present in necroptotic cells [30]. These observations indicated that our sA5-YFP system is capable of detecting other types of PCD than caspase-dependent PCD in vitro.

### sA5-YFP labels specifically PCD cells in a transgenic mouse line

For ex- and in vivo visualization of PCD, we generated a transgenic mouse line expressing sA5-YFP from CAG-sA5-YFP mESCs. Mice were viable and did not display any overt phenotype. Quantitative PCR revealed that the transgene had randomly integrated only once in the genome (Supplementary Fig. 1c).

**Fig. 2:**
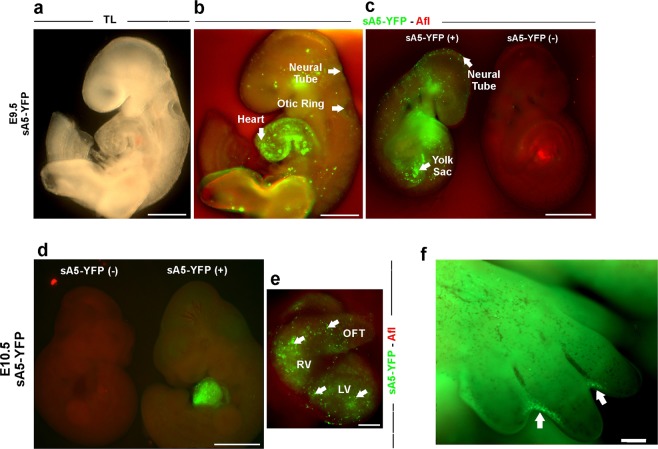
PCD detection in transgenic CAG-sA5-YFP mouse embryos. Macroscopic pictures of WT and transgenic embryos at E9.5 (**a**–**c**) and E10.5 (**d**). Spotty bright green fluorescent signals (white arrows) mark PCD cells in the otic ring, the neural tube, the yolk sac, and the heart of E9.5 and E10.5 mouse embryos. **e** Close-up of a transgenic heart with sA5-YFP expression. **f** Macroscopic picture of a limb from a transgenic E13.5 embryo. Bright green fluorescence signals at the interdigital webbing (arrows) mark PCD cells. TL transmission light, sA5-YFP secreted Annexin V-yellow fluorescent protein, and Afl autofluorescence. Bars are 200 µm (**a**, **b**, **f**), 500 µm (**c**, **d**), and 20 µm (**e**)

To demonstrate that the sA5-YFP system marks cells undergoing PCD in different tissues during mouse development, we isolated transgenic embryos at E9.5–10.5 and screened them under a fluorescence macroscope for bright sA5-YFP^+^ signals. Indeed, in E9.5 embryos this analysis confirmed small, roundish, and bright sA5-YFP^+^ signals in the hindbrain, yolk sac, heart, and otic ring (Fig. 2a–c, white arrows). At E10.5, embryos were less transparent, thereby limiting the macroscopic identification of sA5-YFP^+^ signals in the brain and otic ring. However, in hearts we could detect bright and rounded sA5-YFP^+^ cells with high signal-to-noise ratio indicating accumulation of sA5-YFP in the outer membrane of PCD cells in specific cardiac regions, such as the OFT and both ventricles (Fig. 2d, e, white arrows). At E13.5 we could detect bright sA5-YFP^+^ cells at the interdigital webbing of the limbs (Fig. 2f, arrows), where apoptosis is known to take place at this time point [33].

**Fig. 3:**
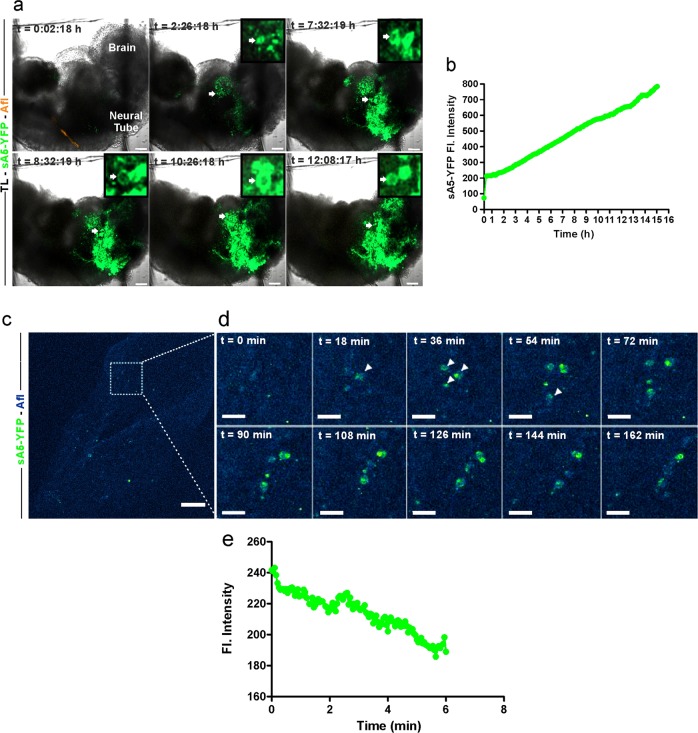
Time-lapse microscopy of PCD in a sA5-YFP embryo and during cranial neural tube closure. **a** Tracking of PCD events in an E8.75 sA5-YFP embryo upon induction of massive cell death by medium starvation (one frame every 5 min over 15 h, z-stack with 4 µm slices; images depicted are from slice #17, boxed images are close-ups from regions with arrows). After 2 h, sA5-YFP^+^ rounded cells (white arrows) appeared. **b** Fluorescence intensity from **a** over time. **c**, **d** Time-lapse microscopy of the brain of an E8 sA5-YFP embryo: the area marked with a white box in **a** is magnified in the subsequent pictures in **d**. After 18 min, the first rounded sA5-YFP^+^ cells (white arrows) appeared; notice that these cells stayed (dancing like) in this area without being engulfed for about 6 h. **e** Quantitation of sA5-YFP fluorescence signals from **d** over time. sA5-YFP secreted human Annexin V-yellow fluorescent protein, TL transmission light, and Afl autofluorescence. Bars are 100 μm (**a**, **c**) and 40 μm (**d**)

Next, as a proof of principle, we wanted to assess the utility of our reporter system to monitor PCD cells in the entire embryo over time using ex vivo confocal time-lapse microscopy. An E8.5 aA5-YFP embryo undergoing serum starvation for 10 h displayed massive cell death, as was evident by the accumulation of sA5-YFP at the membrane of PCD cells (Fig. 3a and video 3). The PCD cells showed an intact ring-like membrane marked by sA5-YFP (Fig. 3a, white arrows in upper panel and close-ups) 6–8 h after PCD induction. After 8 h of live imaging, the embryo started to shrink and displayed massive accumulation of sA5-YFP^+^ cells throughout. In addition, the morphological changes of sA5-YFP labeled cells were accompanied by a constant increase of sA5-YFP fluorescence intensity (Fig. 3b) indicating that our genetic tool is suitable to monitor qualitatively and quantitatively PS externalization and morphological changes of PCD cells in real time in mouse embryos.

### sA5-YFP enables live monitoring of PCD events during embryonic development

**Fig. 4:**
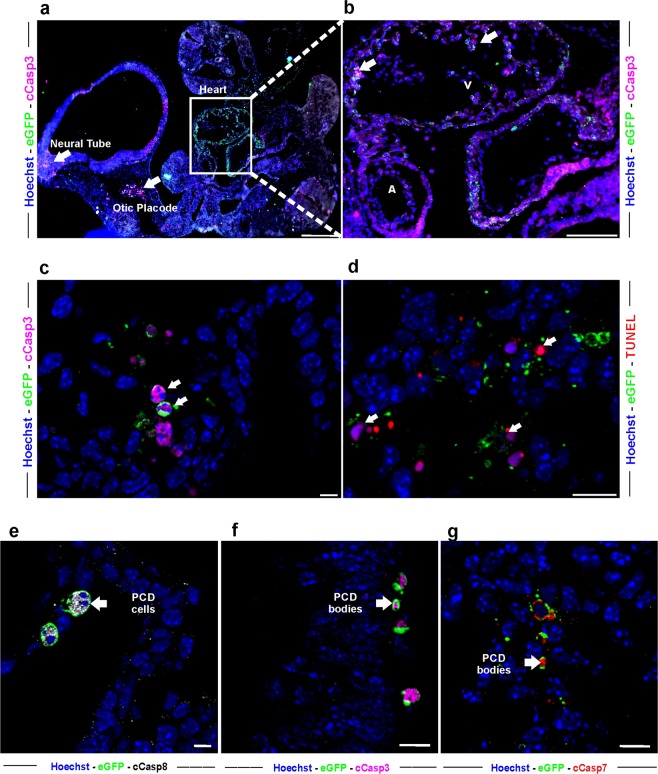
sA5-YFP marks PCD cells at different time points of the PCD cascade in transgenic embryos. **a**, **b** Sagittal section of a transgenic E9.5 embryo showing colocalization of bright sA5-YFP^+^ signals (white arrows) with cCasp3^+^ signals in the heart, the neural tube, and the otic placode. Magnification of the boxed area in **a**. **c**, **d** Sections of the neural tube region demonstrating colocalization of bright sA5-YFP^+^ signals with cCasp3 and to some extent with TUNEL positive signals (white arrows), respectively. **e–g** Sections of sA5-YFP embryos at E10.5 showing colocalization of bright sA5-YFP^+^ particles with cCasp3, 7 and 8. Notice that membranes of cCasp8^+^ cells appeared intact; these cells contained condensed nuclei and a clear YFP^+^ membrane ring indicative for early PCD. Small PCD bodies were found to colocalize with cCasp3 and cCasp7 indicative for intermediate–late PCD. **e** PCD cells marked by sA5-YFP were defined as bigger particles (5–10 µm) that presented condensed nuclei and a cCasp3^+^ signal. **f**, **g** PCD bodies were defined as smaller particles (2–4 µm) without a nuclear signal and YFP^+^ and/or cCasp3^+^. TL transmission light, TUNEL terminal deoxynucleotidyl transferase dUTP nick end labeling, cCasp3 cleaved caspase 3, Hoechst nuclear staining, V ventricle, and A atria. Sections were stained with eGFP antibody after antigen-retrieval treatment to detect sA5-YFP signals. cCasp3,7,8 cleaved caspase 3,7,8. Bars are 200 µm (**a**), 50 µm (**b**), 20 µm (**c**), 10 µm (**d**–**f**), and 2 µm (**g**)

Next, we assessed the specificity and utility of our system for live monitoring PCD cells in the context of normal embryonic development. For this purpose, we imaged the ridges and the midline of the neural tube during fusion, as in both regions PCD is known to occur [34]. Transgenic E8 sA5-YFP embryos were imaged ex vivo using time-lapse confocal microscopy for 10 h (Fig. 3c, d, Video 4, *n* = 3). We observed that within 18 min of live imaging first sA5-YFP^+^ signals appeared in the dorsal ridges of the neural plate and after 38 min bright sA5-YFP^+^ cells displaying PCD morphology became visible (Fig. 4d, white arrowheads). These “dancing PCD cells” corroborated our earlier findings using a different genetic model for live imaging [34]. The cells stayed in these regions, accompanied neural tube closure and slowly fragmented over the imaged time period. The disappearance of the PCD cells was corroborated by a decrease of sA5-YFP fluorescence intensity (Fig. 3e). These data proved that our sA5-YFP tool is suitable for in vivo visualizing PCD cells over time under physiological conditions using live imaging in mouse embryos.

### sA5-YFP detects early–intermediate stages of caspase-dependent PCD during mouse development

In order to define the time frame of the PCD process detected by sA5-YFP more precisely, we performed stainings against cCasp3 and TUNEL in sections of transgenic E9.5 embryos. Approximately one-third of all sA5-YFP^+^ signals colocalized with cCasp3^+^ labeled cells within the otic placode, neural tube, and heart (Fig. 4a, b, white arrows). In addition, most of these sA5-YFP^+^/cCasp3^+^ cells exhibited, as shown by Hoechst counterstaining, nuclear condensation, a hallmark of PCD (Fig. 4c, white arrows). The immunostainings also revealed that mainly very small sA5-YFP^+^ PCD bodies were close to the bright TUNEL^+^ signals (Fig. 4d, white arrows). These data proved that our reporter detects cells undergoing PCD prior to terminal DNA fragmentation, which is marked by TUNEL staining.

We also wondered whether sA5-YFP^+^ signals colocalized with both initiator and effector caspases of the PCD cascade. Immunostainings for cleaved caspase 8, 3, and 7 in sections of E10.5 sA5-YFP embryos revealed colocalization of bright sA5-YFP^+^ signals with all three markers (Fig. 4e–g, white arrows). We noticed that the sA5-YFP^+^ cells co-localizing with cCasp8 (an early marker of caspase-dependent PCD [35]) had a clear ring-like membrane and a condensed nucleus, whereas cCasp8 was distributed throughout the cytoplasm (Fig. 4e). These cells (diameter 5–10 µm) appeared intact and prior to membrane blebbing, thus at the beginning of the PCD cascade, when cells start to shrink and to round up. We defined these cells as “PCD cells” (Fig. 4e). In contrast, sA5-YFP^+^ signals co-localizing with cCasp3 and cCasp7 (both early–intermediate markers of caspase-dependent PCD [35]) labeled PCD bodies (diameter of 2–4 µm) without fragmented nuclei (Fig. 4f, g) underscoring that our sA5-YFP reporter marks cells in early–intermediate PCD (as indicated by sA5-YFP^+^/cCasp3^+^/Hoechst^+^) as well as bodies in intermediate–late PCD (as indicated by sA5-YFP^+^/cCasp3^+^/Hoechst^−^ or sA5-YFP^+^/cCasp3^−^/Hoechst^−^). This implies that our reporter has a broader detection range of the PCD cascade than single endpoint measurements for caspase activation.

### sA5-YFP marks PCD cells and bodies in the developing mouse heart

**Fig. 5:**
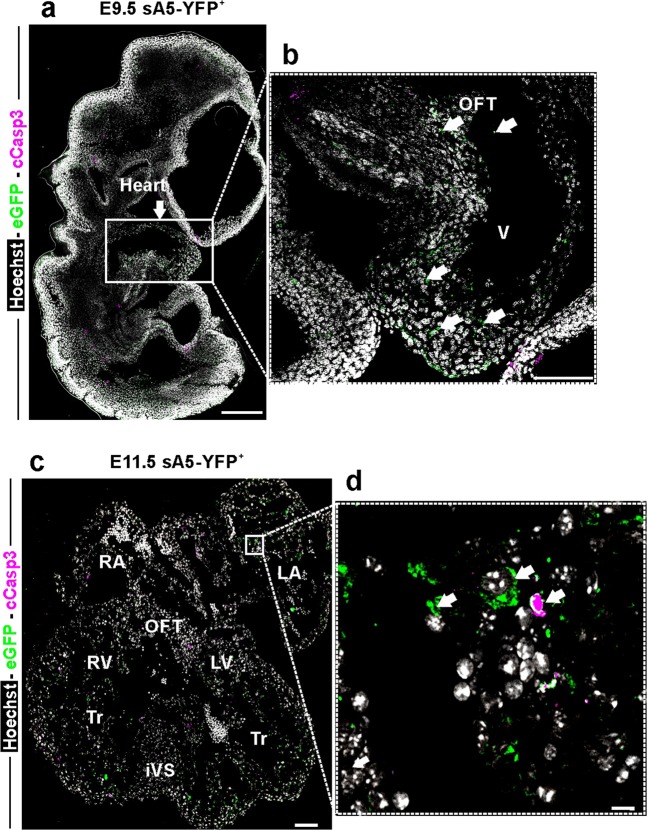
PCD in the mouse heart during early embryonic development. **a**–**d** Sagittal sections of a transgenic embryo at E9.5 and of an E11.5 heart. **b**, **d** Magnification of the boxed areas: bright YFP^+^ signals (white arrows) mark PCD cells or PCD bodies. cCasp3 cleaved caspase 3, Hoechst nuclear signal, V ventricle, OFT outflow tract, RA right atrium, LA left atrium, Tr trabeculae, and iVS intraventricular septum. Bars are 200 µm (**a**), 10 µm (**b**), 100 µm (**c**), and 10 µm (**d**)

**Fig. 6:**
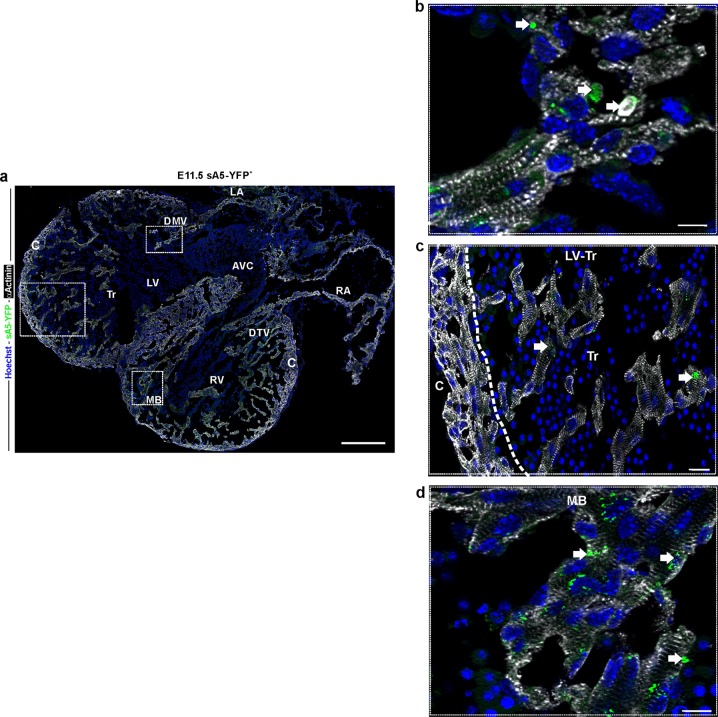
Localization of PCD cells in the E11.5 mouse heart. **a** Frontal section of a transgenic heart at E11.5. Labeled sA5-YFP PCD cells and bodies (white arrows) were found at the trabeculae of both ventricles, in the regions of the developing mitral and tricuspid valves, in the intraventricular septum, and in the moderator band. **b**–**d** Magnifications of the boxed areas in **a** depicting the presence of PCD (sA5-YFP, arrows). Note the striation of cardiomyocytes due to α-actinin staining. Hoechst nuclear signal, sA5-YFP secreted human Annexin V-yellow fluorescent protein, αActinin cardiomyocyte marker, V ventricle, AVC atrioventricular cushion, RA right atrium, LA left atrium, Tr trabeculae, C compact myocardium, MB moderator band (connects the iVS to the anterior papillary muscle), DTV developing tricuspid valve, and DMV developing mitral valve. Bars are 200 µm (**a**), 10 µm (**b**, **d**), and 20 µm (**c**)

**Fig. 7:**
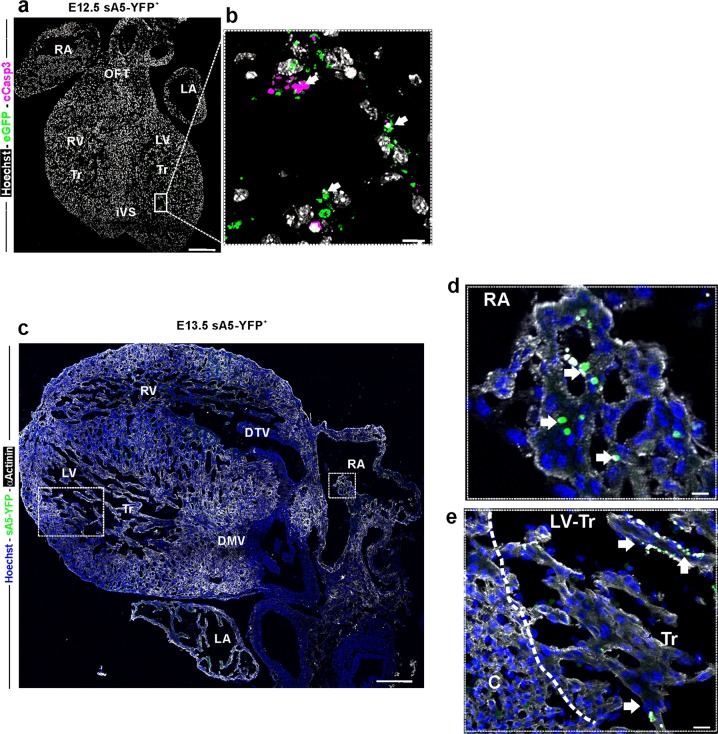
Localization of PCD cells in E12.5 and 13.5 mouse hearts. **a**, **b** Sagittal sections of a transgenic heart at E12.5. Magnification of the boxed area in **a**: bright YFP^+^ signals (white arrows) mark PCD cells or PCD bodies **b**. **c** Frontal sections of a transgenic heart at E13.5. Labeled sA5-YFP PCD cells and PCD bodies (white arrows) were found in the trabeculae of both ventricles, the developing mitral valve, and the right atria. **d**, **e** Magnifications of boxed areas in **a** depicting the presence of PCD (sA5-YFP, arrows). V ventricle, RA right atrium, LA left atrium, Tr trabeculae, DTV developing tricuspid valve, and DVM developing mitral valve. Bars are 200 µm (**a**, **c**) and 20 µm (**b**, **d**, **e**)

There have been few comprehensive studies of PCD in the developing heart in mammals also because of a lack of techniques that can detect the full and ongoing extent of PCD (early-to-late stage), specifically its early phase before DNA fragmentation [10]. Therefore we utilized our genetic PCD reporter to more accurately detect early PCD cells in previously reported areas of cardiac septation in the mouse from E9.5 to E18.5 embryos and postnatal day 2 (P2) hearts. At early embryonic stages (E9.5–E10.5) sA5-YFP^+^ labeled cells were mainly localized to the ventricular myocardium and OFT (Fig. 5a, b, white arrows). Later, at E11.5, sA5-YFP labeled cells could be detected in the atria (Fig. 5c, d), the trabeculae of both ventricles, the developing mitral and tricuspid valves, the interventricular septum, and at the moderator band (connects the iVS to the anterior papillary muscle) (Fig. 6a–d, white arrows). Interestingly, we also found that sA5-YFP^+^ PCD bodies were close to cCasp3^+^ signals (Supplementary Fig. 3a, b, white arrows) in the OFT indicating that cells in this area could have been already engulfed or were in a late stage of PCD. At E12.5, there was presence of PCD cells and bodies in the trabecular region of the left ventricle (Fig. 7a, b, white arrows, and Supplementary Fig. 4a–c, white arrows). At this stage, it was also possible to identify cells at intermediate–late stage of PCD at the apex of the iVS, in the compact zone of the ventricular wall, and in the OFT. In summary, we detected PCD cells and bodies in the developing valves, OFT, intraventricular septum, compact myocardium of the ventricular chambers, and in the atrial septum in E10.5–12.5 sA5-YFP mouse hearts consistent with previous work. Importantly, our genetic system enabled us to identify new and unexpected cardiac regions, where early–intermediate PCD took place. Firstly, we detected PCD in the primitive ventricle, the initial ventricular trabeculations, and in the OFT of E9.5 hearts (Fig. 5a, b). In addition, at E13.5 we noticed sA5-YFP^+^ cells in the left trabecular region, in the right atria, and in the tricuspid valve (Fig. 7c–e, white arrows), which were overlooked in previous work [11, 12]. Our results suggest that PCD might be relevant to trabeculae maturation. At later stages (E15.5–E18.5), the PCD events were sporadically found in the ventricles and close to the mitral and tricuspid valves (Supplementary Fig. 5a, b, white arrows). Finally, the presence of PCD cells and bodies in P2 hearts was scarce (Supplementary Fig. 6a, b, white arrows).

### sA5-YFP enables live monitoring of PCD events during heart development

As the sA5-YFP system is optimally suited for live imaging, we monitored E9 transgenic embryos for PCD events in the developing heart ex vivo. Over a time course of 20 h we observed an increase of sA5-YFP in the membrane of dying cells in the ventricle, the epicardium, and the OFT (Supplementary Fig. 7a, video 5). After 6 h, the first rounded cells and PCD bodies (Supplementary Fig. 7a, white dotted circles) appeared in the right ventricle, left epicardium, and OFT. Furthermore, it was also noticed that some PCD cells appeared and disappeared over time (yellow box in Supplementary Fig. 7a) indicating that they could have been phagocytized by macrophages or other engulfing cells. This was supported by the increase and drop in fluorescence in synchrony with the appearance and disappearance of PCD cells (Supplementary Fig. 7b). Some of the sA5-YFP^+^ cells preserved their roundish morphology over the imaging period (Supplementary Fig. 7a, arrows).

### sA5-YFP^+^ signals identify early–intermediate PCD and peak at early stages of heart organogenesis

To further specify stage and extent of PCD in cardiac tissue we compared the sA5-YFP^+^ signals of our transgenic system with cCasp3 and TUNEL stainings. Quantification of PCD cells and bodies in E9.5–E17.5 hearts revealed a better recognition of PCD events by sA5-YFP compared with either cCasp3 or TUNEL (Supplementary Fig. 8a–c, *n* = 2–3). As observed in E9.5 embryos, the least overlap was between TUNEL^+^ and sA5-YFP^+^ cells, indicating recognition of mainly early–intermediate and only limited late PCD events by sA5-YFP. At all analyzed stages sA5-YFP signals displayed overlap between both cCasp3^+^ cells and PCD bodies and TUNEL^+^ cells. However, according to our criterion of a PCD body, which was defined as having no DNA, there were no TUNEL positive PCD bodies, as TUNEL labels fragmented DNA. In summary, sA5-YFP recognized the most PCD events (cells and bodies), followed by cCasp3 (cells and bodies) and TUNEL staining (only cells).

**Fig. 8:**
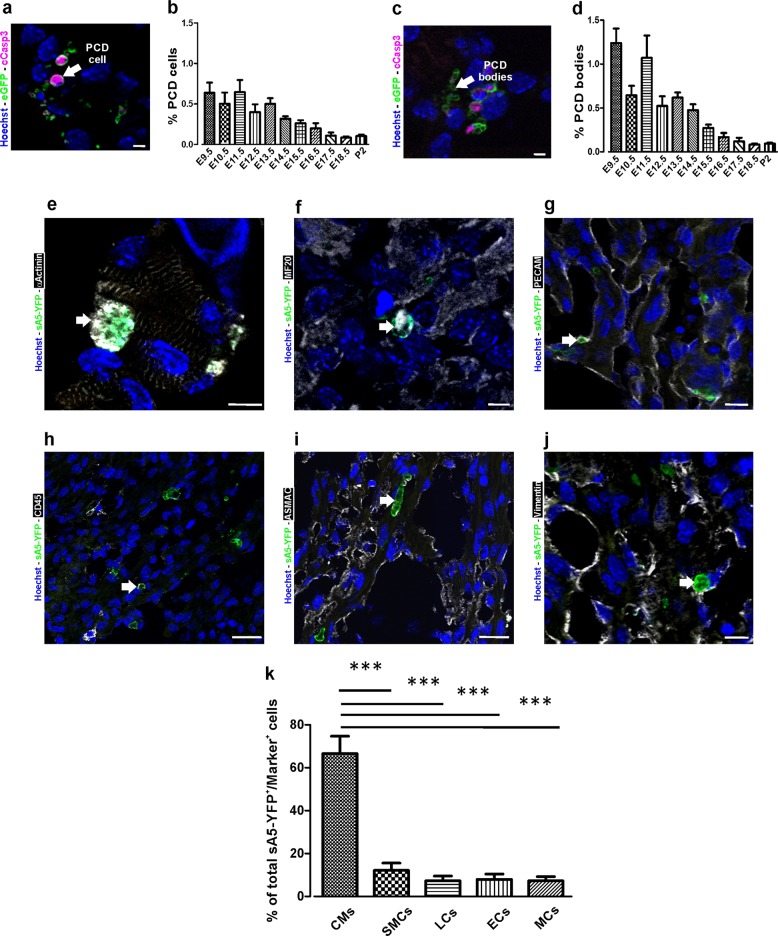
Identification and quantitation of the different cell types undergoing PCD during heart development. **a** Sagittal section of a transgenic heart at E12.5 depicting PCD cells. PCD cells were defined by condensed nuclei, a clear ring-like membrane sA5-YFP accumulation, and/or cCasp3^+^ signal. **b** Quantitation of PCD cells (white arrow in a). **c** Sagittal section of a transgenic heart at E12.5 depicting bodies of PCD cells. PCD Bodies were defined as smaller particles (2–4 µm) without nuclear signal and positive for either sA5-YFP or cCasp3 or both. **d** Quantitation of PCD bodies (white arrow in **c**). **e**–**j** Co-stainings in sections of transgenic embryonic hearts (E13.5) stained for cardiomyocyte (CMs, α-actinin, and MF20), endothelial cell (ECs, PECAM), leukocyte (LCs, CD45), smooth muscle cell (SMCs, ASMAC), and mesenchymal cell (MCs, vimentin) markers. **k** Quantitation of the different cell types that undergo PCD during heart development. Hoechst nuclear staining and sA5-YFP secreted human Annexin V-yellow fluorescent protein. Bars are 5 µm (**a**, **c**, **e**, **f**), 10 µm (**g**, **j**), and 20 µm (**h**, **i**). Data are shown as mean ± s.e.m.,***P < 0.001 ANOVA with Bonferroni post hoc test; *n* = 7–13

To assess the extent of PCD during heart development, we quantified both early (Fig. 8a) and intermediate (Fig. 8b) PCD events in sA5-YFP hearts from E9.5 to P2 (Fig. 8b, d, *n* = 3). We found that PCD peaked at early stages of heart organogenesis (E9.5–E14.5) and decreased from E15.5 onward (Fig. 8h, j), as suggested in earlier reports [36, 37]. In detail, the percentage of total cells in the early phase of PCD (indicated by sA5-YFP^+^/cCasp3^+^/Hoechst^+^) was ≤0.7% of all cardiac cells from E9.5 to E14.5 and decreased to ≤0.25% from E15.5 to P2 (Fig. 8b). The percentage of PCD bodies (indicated by sA5-YFP^+^/cCasp3^+^/Hoechst^-^) was ≤1.4% from E9.5 to E14.5, and ≤0.27% from E15.5 to P2 (Fig. 8d). These numbers suggest that the cells had already undergone PCD at even earlier stages of heart development (i.e., E7.5–E8.5).

Besides the overall PCD rates in different regions of the developing heart, we were also interested to determine which cardiac cell types undergo PCD. For this, we performed co-stainings of sections from E13.5 sA5-YFP hearts with typical markers for cardiomyocytes (α-actinin and MF20), endothelial cells (PECAM), leukocytes (CD45), and smooth muscle cells (ASMAC). Our analysis revealed a strong colocalization between cardiomyocytes and sA5-YFP^+^ signals (Fig. 8e, f) with ~66% of the sA5-YFP^+^ cells being cardiomyocytes (Fig. 8e, f, k), and only 8% endothelial cells (Fig. 8g, k), 7% leukocytes (Fig. 8h, k), 12% smooth muscle cells (Fig. 8i, k), and 7% mesenchymal cells (Fig. 8j, k). Thus, our data suggest that the embryonic, but not the P2 heart depends on PCD within the context of morphogenesis and that particularly cardiomyocytes are involved in this process (Fig. 8k).

## Discussion

A major challenge in the field of PCD is its accurate detection, quantification, and observation over time ex- and in vivo, particularly in the mammalian heart [38, 39]. In fact, it is still not clear, where and to which extent PCD occurs during embryonic development. We met this challenge by establishing a genetic PCD detection system in transgenic mice consisting of ubiquitously expressed sA5 fused to YFP. The sA5-YFP colocalizes with active forms of pro-caspases (caspase 8), effector caspases (caspase 3 and 7), and with TUNEL staining. Since cCasp3 is a key marker of early–intermediate [27, 28], and TUNEL of late PCD our data suggest that based on its overlap with these two markers sA5-YFP labels early–intermediate PCD stages.

Furthermore, PCD bodies of sA5-YFP labeled cells can be detected before they are engulfed proving that the sA5-YFP reporter allows the visualization of cells in different stages (early–intermediate–late) of PCD. Both PCD cells and PCD bodies can be quantified by measuring the sA5-YFP intensity in real time or by detecting round and bright sA5-YFP^+^ signals in fixed tissue. We were also able to detect typical morphological and biochemical features of PCD cells, namely PS exposure marked by sA5-YFP, nuclear condensation labeled by Hoechst staining, and cCasp3 activation at the same time. This fulfills the criteria of using at least two biochemical methods besides morphological aspects to define specifically the type of PCD [28]. Because of the combination of PCD markers, we could clearly distinguish between early (PCD cells with condensed nuclei) and intermediate–late stages (PCD bodies without nuclei) of PCD.

The main advantage of our sA5-YFP system is the detection of PCD during mouse embryonic development by direct visualization of bright sA5-YFP^+^ cells without further processing. The fluorescent signal remains stable after tissue fixation allowing co-staining tissues with other PCD and cell type markers. Importantly, it is one of the few live imaging tools enabling the ex- and in vivo visualization of PCD in mammals, as underscored by the monitoring of PCD cells during neural tube closure [35].

We have explored in detail PCD events in the developing mouse heart and detected PCD cells and bodies in different areas of E11.5–E13.5 sA5-YFP hearts, confirming previous research [11, 12, 36, 40]. Importantly, we also observed PCD cells and bodies in the primitive ventricle at E9.5 and in the lining of the trabeculae of both ventricles from E10.5 to E14.5 in sA5-YFP hearts. This could be due to the detection of early PCD cells by sA5-YFP prior to engulfment. Interestingly, these early–intermediate PCD events coincide with episodes of trabeculae formation, differentiation, remodeling, and compaction, suggesting that PCD could play an important role for chamber maturation. Trabeculations are first evident after cardiac looping around E9.5–E10.5 in mouse hearts [41]. First signs of trabeculae formation were observed in sA5-YFP embryos at E9.5 and coincided with the presence of bright sA5-YFP^+^ cells and bodies throughout the ventricle. During later developmental stages (E11.5–E13.5) numerous labeled sA5-YFP^+^ PCD cells and bodies were mainly found in the ventricular trabeculae close to the developing valves and in the non-compact zone. From E14.5 onward, sA5-YFP^+^ PCD cells were scarcely found in the compact myocardium suggesting that PCD is switched off as the heart grows older in agreement with other studies [42]. In line with these results, we demonstrated that the labeled sA5-YFP^+^ PCD cells and bodies, found in the ventricular chambers, are preferentially cardiomyocytes, as suggested earlier [43]. These findings imply a possible role of cardiomyocyte PCD in ventricular differentiation and trabeculation, whose perturbation could underlie pathological conditions.

In addition, we provide a complete map of the incidence of early–intermediate PCD in embryonic and neonatal mouse hearts from E9.5 to P2 showing that early PCD peaks at early–intermediate stages of heart development (E9.5–E14.5) and decreases from E15.5 onward. Our observations match the results obtained with a secA5-YFP transgenic zebrafish line [9], in which the brightest TUNEL^+^ signals were not positive for YFP and showed no nuclear staining. These cells were most likely in the final stage of PCD, where DNA fragmentation is massive and PCD cells do not have a defined cell membrane and nucleus. The percentage of PCD present in the embryonic heart is significantly higher than in the adult heart (≤1.4% vs. 0.001%, respectively) [44, 45]. A precise estimation of PCD rates is critical, as a slight increase in the PCD cell number could lead to heart failure. For instance, Kitsis et al. demonstrated that PCD rates of 0.023% are sufficient to induce a lethal, dilated cardiomyopathy in adult mouse hearts [45] and these levels of PCD are four-to ten fold lower than the one reported in failing human hearts [46, 47]. Although mice with a deletion of both the Bax and Bak pro-apoptotic genes have no obvious heart phenotype [48] it is difficult to draw any conclusions for cardiac biology, as the knockout of these two PCD-related genes has been shown to result in the activation of alternative, non-apoptotic cell death pathways in various tissues [49–54].

In conclusion, the CAG-sA5-YFP system marks PCD cells in different tissues and cell types, in vitro, ex vitro, in vivo, and in real time. This makes it feasible to explore the role of PCD in mouse heart development by crossing it with mouse models for cardiac malformations.

## Supplementary information

Supplementary figures and video legends

Supplementary figures

Author contribution

Video 1

Video 2

Video 3

Video 4

Video 5

Video 6
