# Supplementary material for: In vivo detection of programmed cell death during mouse heart development
Source: Cell Death Differ. 2019 Sep 30;27(4):1398–414. doi: 10.1038/s41418-019-0426-2 (PMC7205869; doi:10.1038/s41418-019-0426-2)
Supplement: Supplementary file 1 — Supplementary figures and video legends [file 41418_2019_426_MOESM1_ESM.docx]

# SUPPLEMENTARY FIGURES

**Supplementary Fig. 1.** **Generation of the CAG-sA5-YFP expression cassette**.

(a) Scheme of the cloning strategy for the generation of the CAG-sA5-YFP plasmid. (b) Scheme for the detection of PCD by binding of Annexin V to PS. During PCD, PS residues are translocated to the outer leaflet of the cell membrane where they are bound by annexin V with high affinity in a Ca^2+^-dependent manner. (c) Determination of copy number of the sA5-YFP transgene by qPCR. Three sA5-YFP mice were compared to a control mouse line with one transgene integration^1^.

**Supplementary Fig. 2. Characterization of transgenic sA5-YFP mESCs, and inhibition of caspase activity in sA5-YFP mESCs.**

(a) Brightfield (left panel) and epifluorescence (right panel) pictures of transgenic mESCs clones stably expressing sA5-YFP. (b) Transgenic sA5-YFP mESCs were incubated with the pan-caspase inhibitor (zVAD-fmk) at 50 µM 2 h before cell death induction with 1% DMSO. After 24 h Casp-3 activity was clearly inhibited and bright sA5-YFP^+^ signals (arrows and close-up) could still be observed after inhibition indicating that the sA5-YFP reporter also detects caspase-independent programmed cell death such as necroptosis., n=3. BF = brightfield, cCasp3 = cleaved caspase 3 and sA5-YFP = secreted human Annexin V-yellow fluorescent protein. Bars are 50 µm (a) and 20 µm (b).

Supplementary Fig. 3. Localization of PCD cells in the mouse heart at E11.5.

(a) Frontal section of a transgenic heart at E11.5. sA5-YFP^+^ (eGFP staining) PCD cells and PCD bodies were found at the trabeculae of both ventricles, in the developing valves, and in the OFT. (b) Magnifications of the OFT (boxed area) in (a) reveals cells undergoing apoptosis (arrows). Hoechst = nuclear signal, eGFP = enhanced green fluorescent protein, cCasp3 = cleaved caspase 3, LV = left ventricle, RV = right ventricle, OFT = outflow tract, RA = right atrium, LA = left atrium, Tr = trabeculae, C = compact myocardium, iVS = intraventricular septum, DMV = developing mitral valve, and DTV = developing tricuspid valve. *Sections were stained with an eGFP antibody after antigen retrieval treatment to detect the sA5-YFP signals. Bars are 200 µm (a), and 20 µm (b).

Supplementary Fig. 4. Localization of PCD cells in the mouse heart at E12.5.

(a) Frontal section of a transgenic heart at E12.5: sA5-YFP^+^ (eGFP staining) PCD cells and bodies were found at the trabeculae of both ventricles and in the OFT. (b-c) Magnifications of the heart regions marked by boxed areas in (a) depict the presence of PCD (arrows). Hoechst = nuclear signal, eGFP = enhanced green fluorescent protein, cCasp3 = cleaved caspase 3, LV = left ventricle, RV = right ventricle, OFT = outflow tract, RA = right atrium, LA = left atrium, Tr = trabeculae, iVS = intraventricular septum, C = compact myocardium. *Sections were stained with eGFP antibody after antigen retrieval treatment to detect the sA5-YFP signals. Bars are 200 µm (a), 10 µm (b), and 50 µm (c).

Supplementary Fig. 5. Localization of PCD cells in the mouse heart at E18.5.

(a) Frontal section of a transgenic heart at E18.5. sA5-YFP^+^ PCD cells and bodies were found in the ventricles, close to the mitral and tricuspid valves, and atria. (b) Magnifications of the heart region marked by boxed area in (a) depict the presence of PCD (arrows). Hoechst = nuclear signal, eGFP = enhanced green fluorescent protein, cCasp3 = cleaved caspase 3, LV = left ventricle, RV = right ventricle, RA = right atrium, LA = left atrium, Tr = trabeculae, C = compact myocardium. *Sections were stained with eGFP antibody after antigen retrieval treatment to detect the sA5-YFP signals. Bars are 200 µm (a), and 10 µm (b).

Supplementary Fig. 6. Localization of PCD cells in P2 mouse hearts.

(a) Frontal section of a transgenic heart at P2. sA5-YFP^+^ PCD cells and bodies (arrows) were scarcely found in the epicardium. (b) Magnification of the heart region marked by the boxed area in (a) depicts the presence of PCD(arrows). RA = right atrium, LA = left atrium, LV = left ventricle, RV = right ventricle, cCasp3 = cleaved caspase 3. *Sections in (a,b) were stained with eGFP antibody after antigen retrieval treatment to detect the sA5-YFP signals. Bars are 500 µm (a) and 20 µm (b).

Supplementary Fig. 7. Long term observation of physiological cell death in a sA5-YFP embryonic heart at E9 and isolated E8.5 sA5-YFP embryos.

(a) Long term observation of an E9 sA5-YFP embryonic heart. An increase in accumulation of sA5-YFP in the membrane of dead cells was observed in the ventricle, epicardium and outflow tract of a transgenic sA5-YFP embryo (white dotted circles). After 1 h, the first rounded cells and apoptotic bodies (yellow square) appeared in the outflow tract. Notice that cells marked with an arrow stayed there during the whole time of imaging. Time-lapse images were recorded every 10 min, Z-stack slices were taken and the images in this figure are maximum projections. (b) Fluorescence intensity of the region marked in the yellow box in (a). (c) Macroscopic pictures of E8.5 sA5-YFP chimeric embryos expressing sA5-YFP. Bright green fluorescent signals (white arrows) show typical PCD features. Epi. = epicardium, OFT = outflow tract, RV = right ventricle, sA5-YFP = secreted Annexin V-yellow fluorescent protein, Afl = autofluorescence. Bars are 100 µm (a) and 200 µm (c).

**Supplementary Fig. 8. Quantification of cCasp3^+^, TUNEL^+^, and sA5-YFP^+^ cells and bodies during embryonic heart development.**

(a,b) Quantification of cCasp3^+^, sA5-YFP^+^, and cCasp3^+^/sA5-YFP^+^ cells (containing a nucleus) (a) and bodies (without nucleus) (b), (n=3). (c) Quantification of TUNEL^+^, sA5-YFP^+^, and TUNEL^+^/sA5-YFP^+^ cells (containing a nucleus). n=2-3. (d) Section displaying a part of the trabecular layer of the left ventricle of a transgenic heart at E11.5. sA5-YFP^+^ (eGFP staining) PCD cells and PCD bodies (arrows) and TUNEL^+^ (red) PCD cells were detectable. (e) Magnifications of the boxed area in (d) reveal cells undergoing PCD (arrows). Hoechst = nuclear signal, eGFP = enhanced green fluorescent protein, TUNEL = Terminal deoxynucleotidyl transferase dUTP nick end labeling. *Sections were stained with an eGFP antibody after antigen retrieval treatment to detect the sA5-YFP signals. Bars are 20 µm (d), and 5 µm (e).

**Videos 1-2. Time course of PCD cells in transgenic sA5-YFP (video 1) and non-transgenic G4-mESCs (video 2) upon PCD induction.**

Transgenic sA5-YFP mESCs were treated with 40 µM 4-OHT for 24 h. G4-mESCs were pre-incubated for 30 min with 1 µM of the commercial Annexin V-FITC probe immediately before PCD induction. PCD cells were characterized by accumulation of Annexin V-FITC in their membrane resulting in a bright green fluorescent signal and a roundish morphology. Time-lapse images were recorded every 5 min., frame rate: 5 frames per s. Bar is 20 µm.

**Video 3. *Live* imaging of a transgenic E8.75 sA5-YFP^+^ embryo after induction of massive PCD.**

A transgenic E8.75 sA5-YFP embryo was dissected from the uterus and its yolk sac. It was placed sagittally into holes in a layer of 2% low temperature-melting agarose contained in an imaging chamber. The embryo was observed with an inverted confocal microscope at 37°C and 5% CO_2_. Massive cell death was induced by medium starvation. A clear increase in accumulation of sA5-YFP in the membrane of dying cells throughout the whole embryo was observed. After 2 h, the first rounded cells and blebs (white arrows) appeared. Time-lapse images were recorded every 6 min. for 15 h, 33 z-stack slices of 4 µm were taken. The video is taken from z-stack #17; frame rate: 5 frames per s. Bar is 100 µm.

**Video 4. *Live* imaging of sA5-YFP^+^ PCD cells during neural tube closure.**

A transgenic E8.0 sA5-YFP embryo was imaged with a confocal microscope. As the neural tube closes, bright sA5-YFP PCD cells appeared fragmented and disappeared from the hindbrain region over time. Time-lapse images were recorded every 3 min for 10 h. 20 z-stack slices of 10 µm were taken. Bar is 100 µm.

**Video 5 - 6. *Live* imaging of PCD in a transgenic E9 sA5-YFP^+^ heart.**

The heart of a transgenic E9.0 sA5-YFP^+^ embryo was observed with an inverted confocal microscope. (Video 5) A clear increase in accumulation of sA5-YFP in the membrane of dying cells was observed in the ventricle, epicardium and outflow tract. After 1 h, the first rounded cells and blebs (white arrows) appeared. (Video 6) Close-up of the outflow tract region marked in the green box. Time-lapse images were recorded every 10 min for 20 h. Frame rate: 7 frames per s. Z-stack slices were taken. Images in this Fig. are a maximum projection of all z-stacks. Bar is 100 µm.

References

1. Hesse M, Raulf A, Pilz GA, Haberlandt C, Klein AM, Jabs R*, et al.* Direct visualization of cell division using high-resolution imaging of M-phase of the cell cycle. *NatCommun* 2012, **3:** 1076.
