# Supplementary material for: In vivo detection of programmed cell death during mouse heart development
Source: Cell Death Differ. 2019 Sep 30;27(4):1398–414. doi: 10.1038/s41418-019-0426-2 (PMC7205869; doi:10.1038/s41418-019-0426-2)

Supplementary Figure 1

a

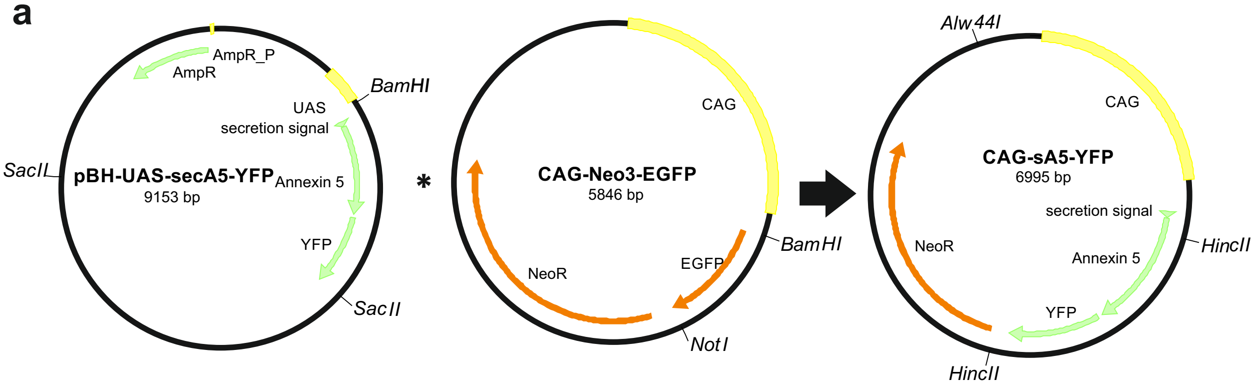

b

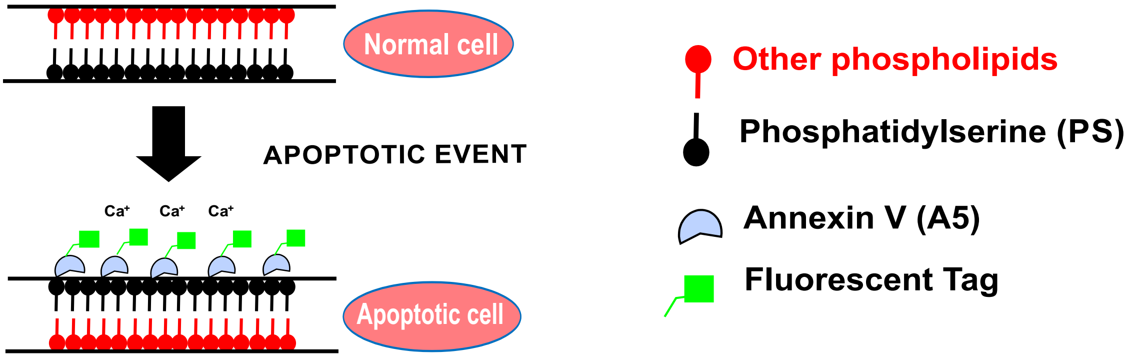

c

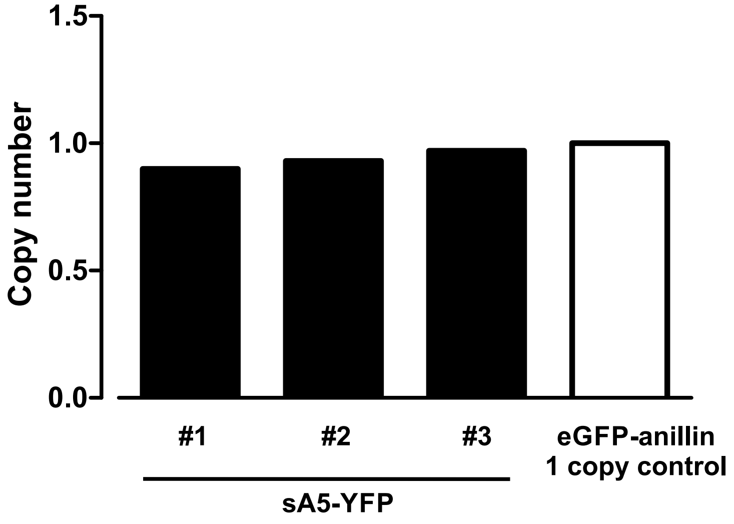

Supplementary Figure 2

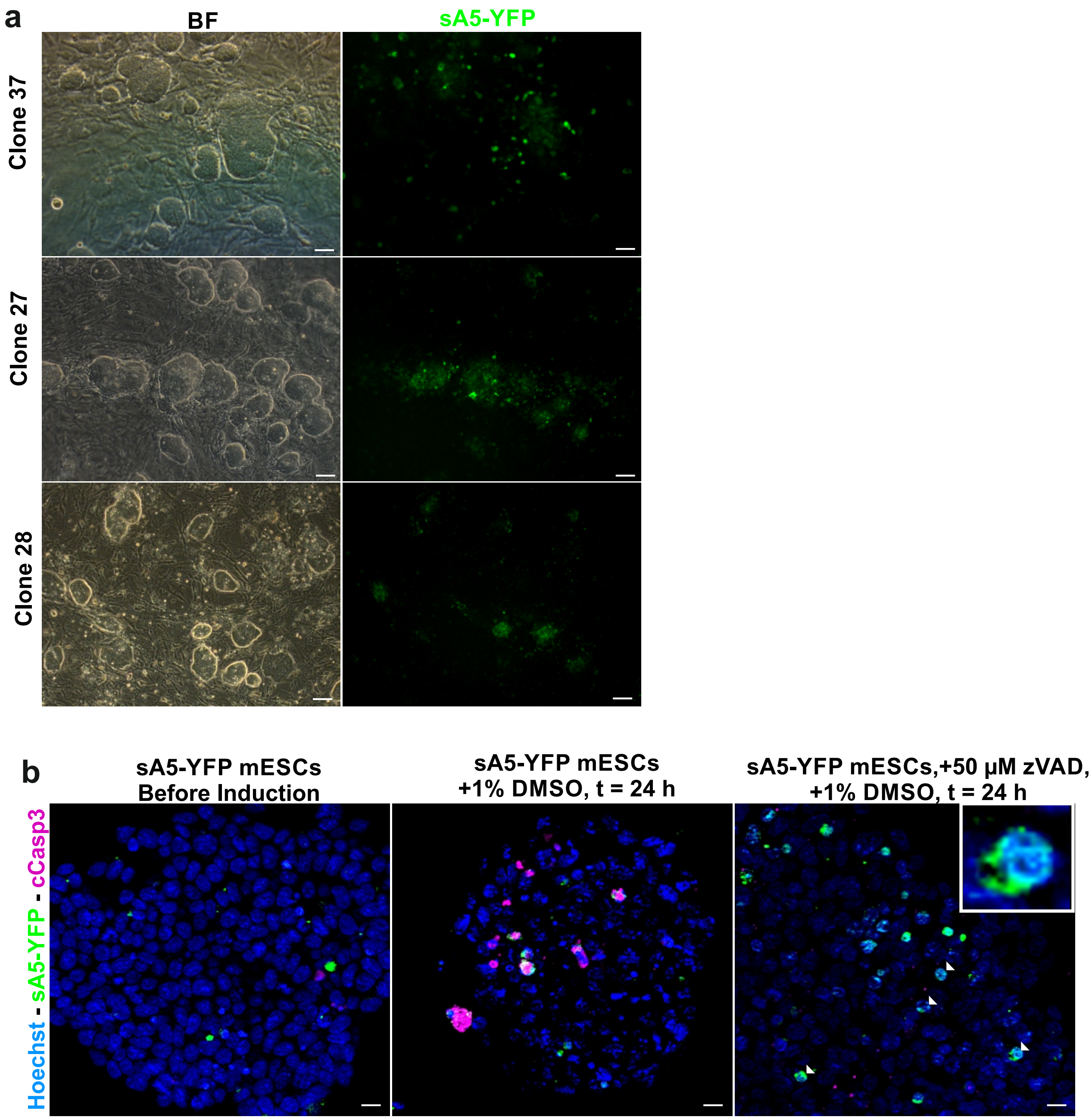

Supplementary Figure 3

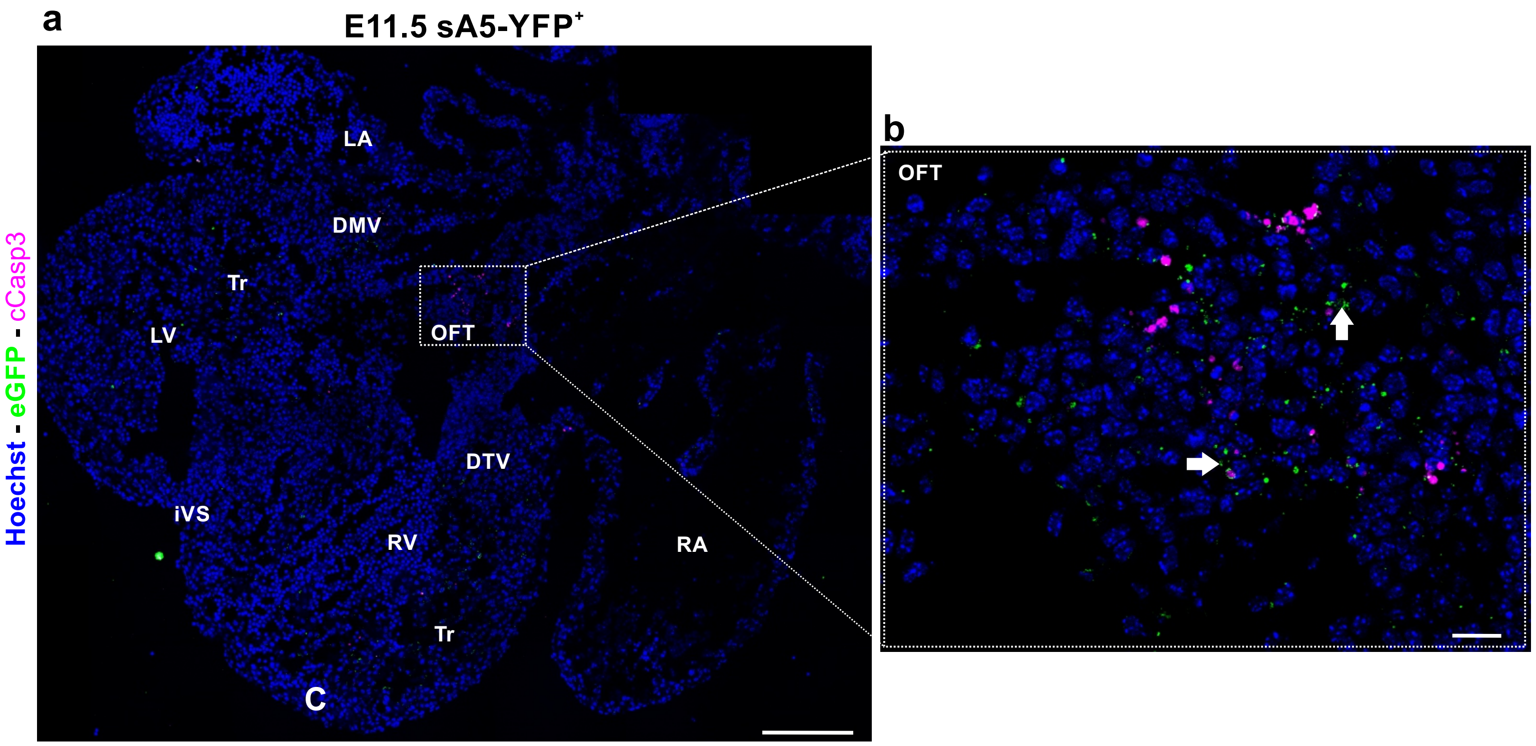

Supplementary Figure 4

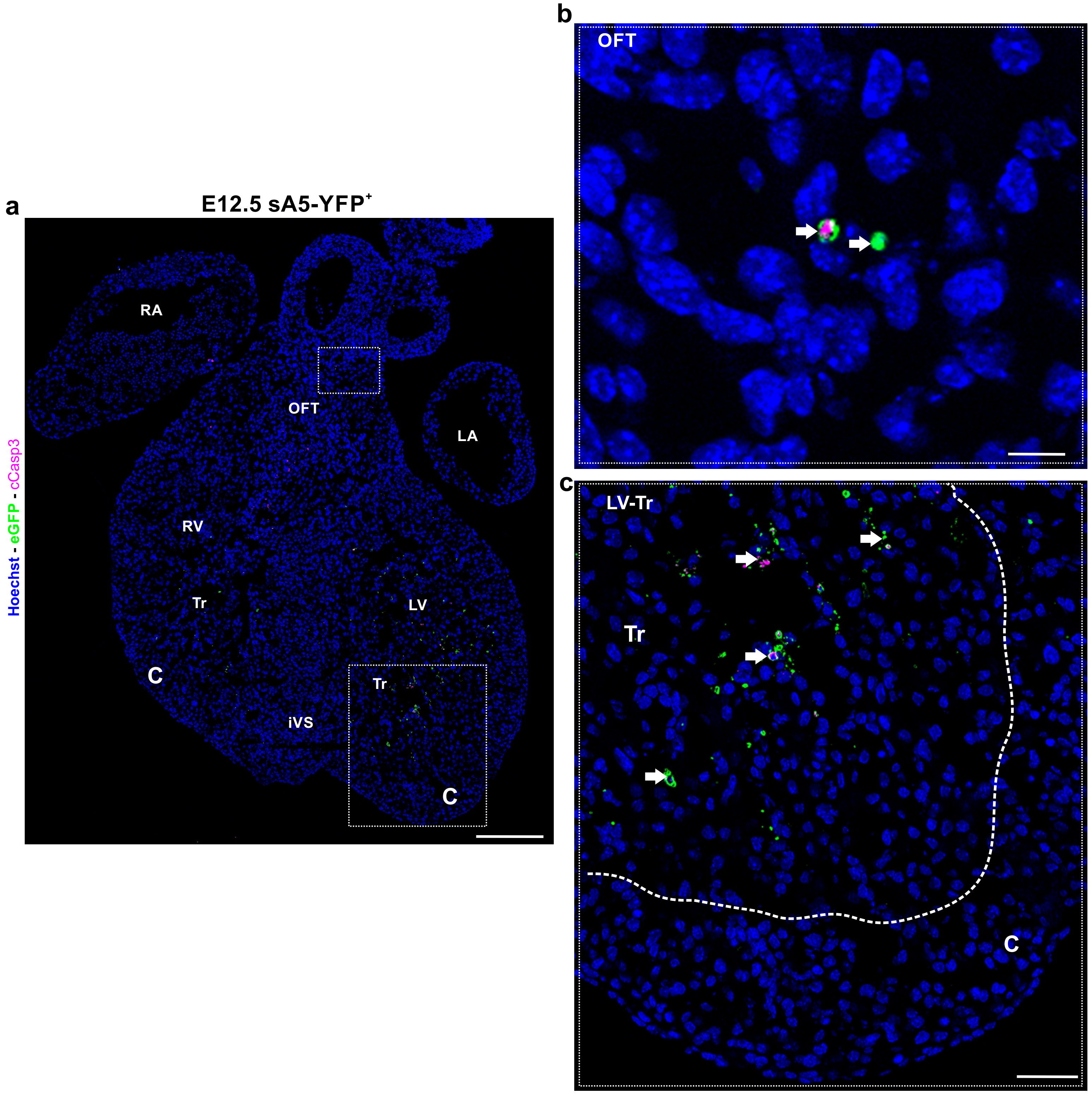

Supplementary Figure 5

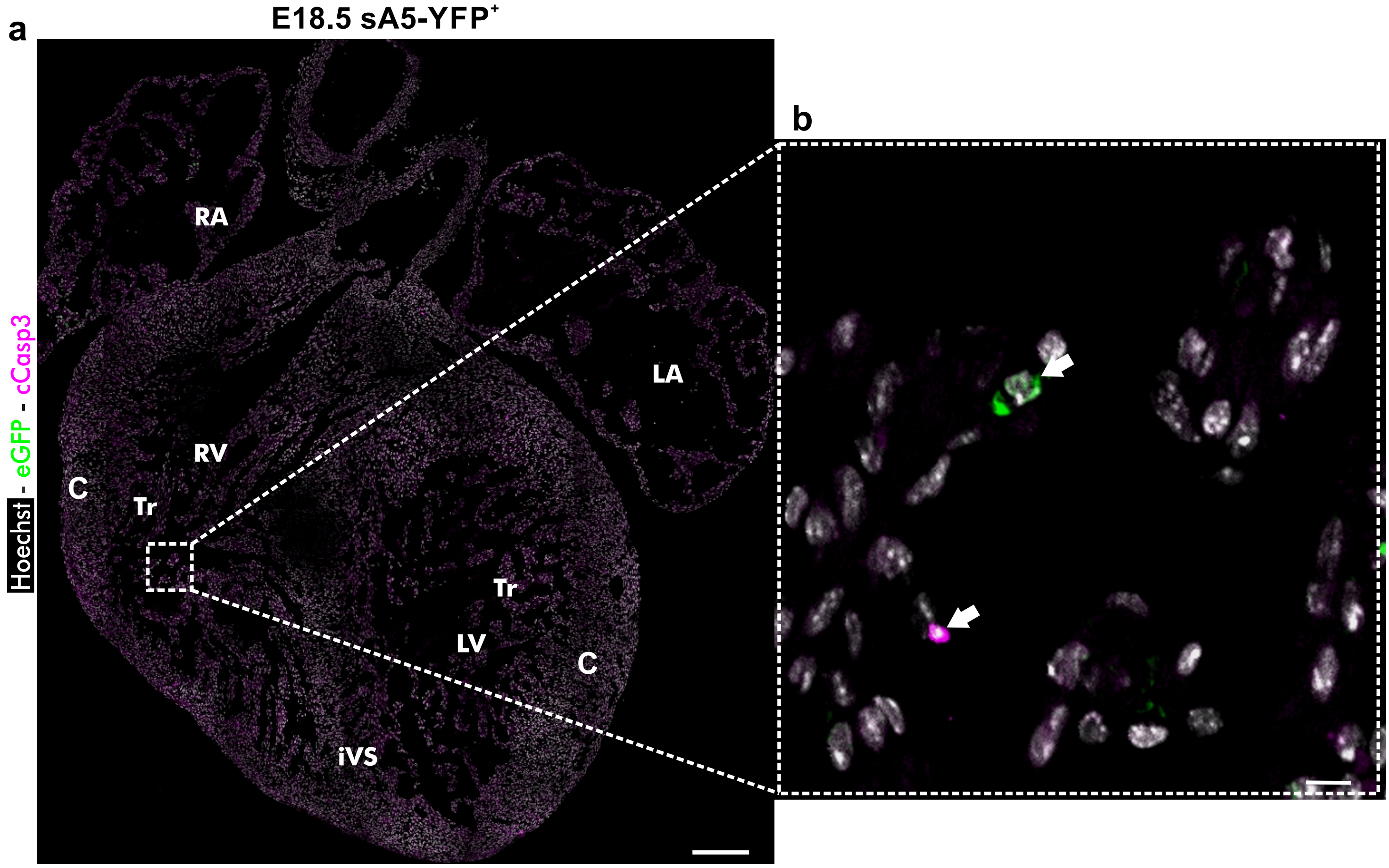

Supplementary Figure 6

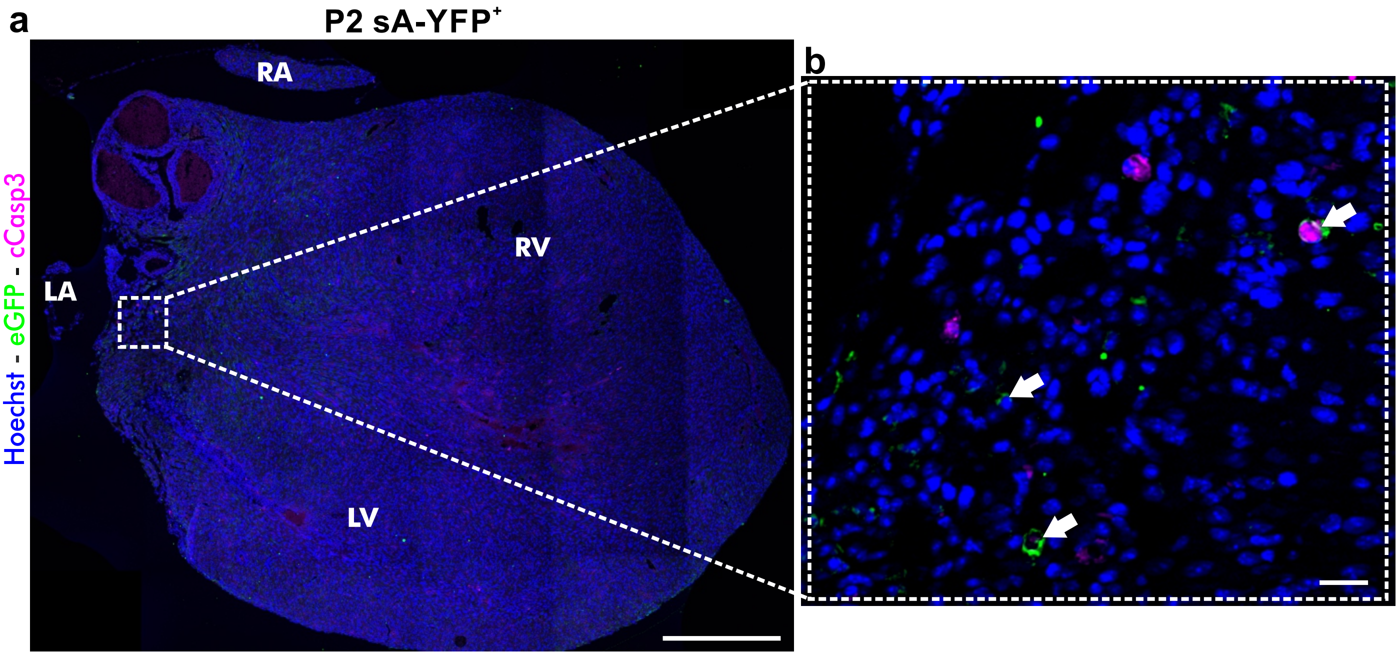

Supplementary Figure 7

a

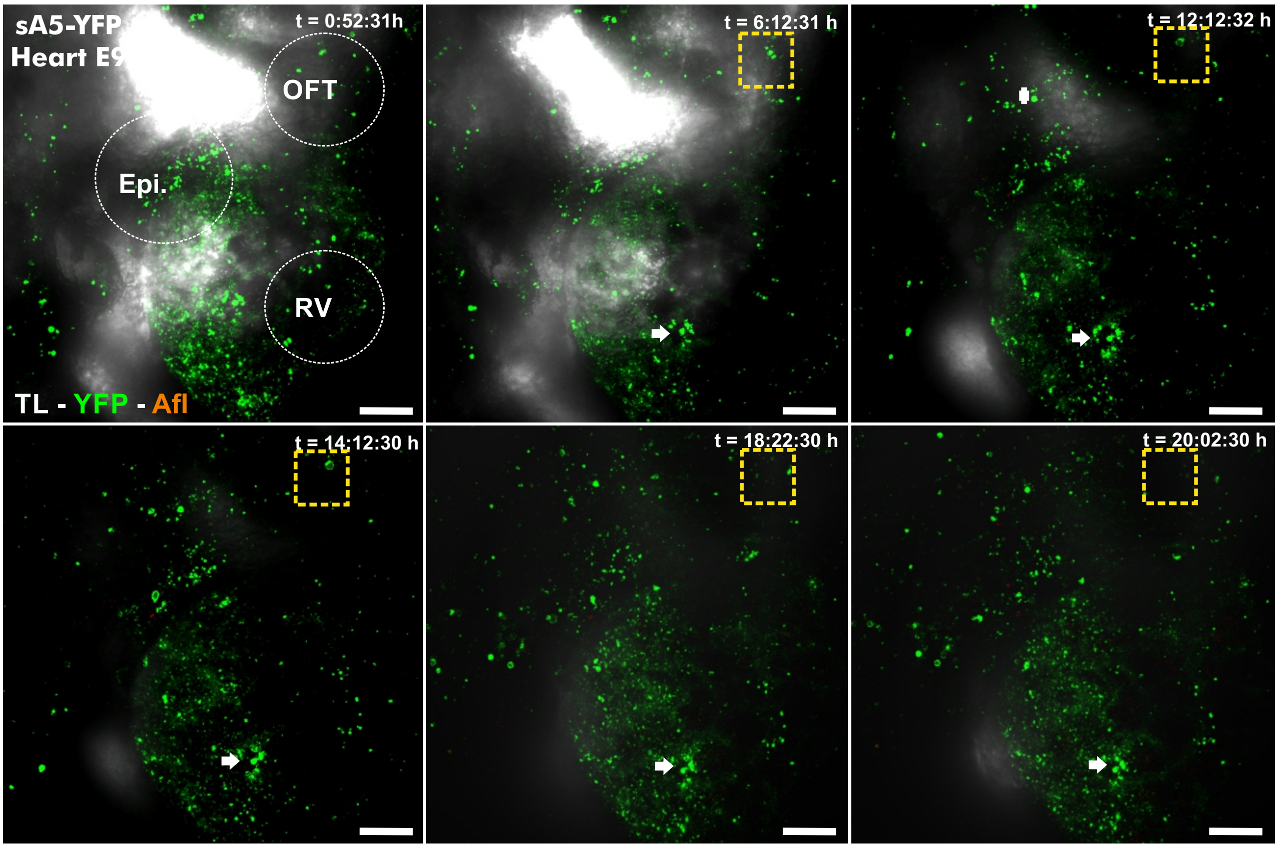

b

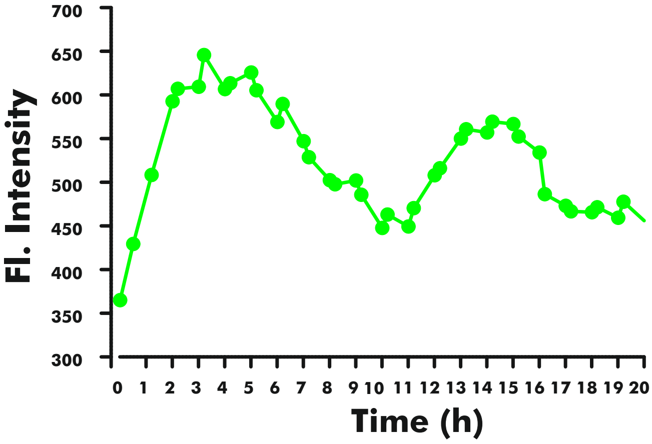

Supplementary Figure 8

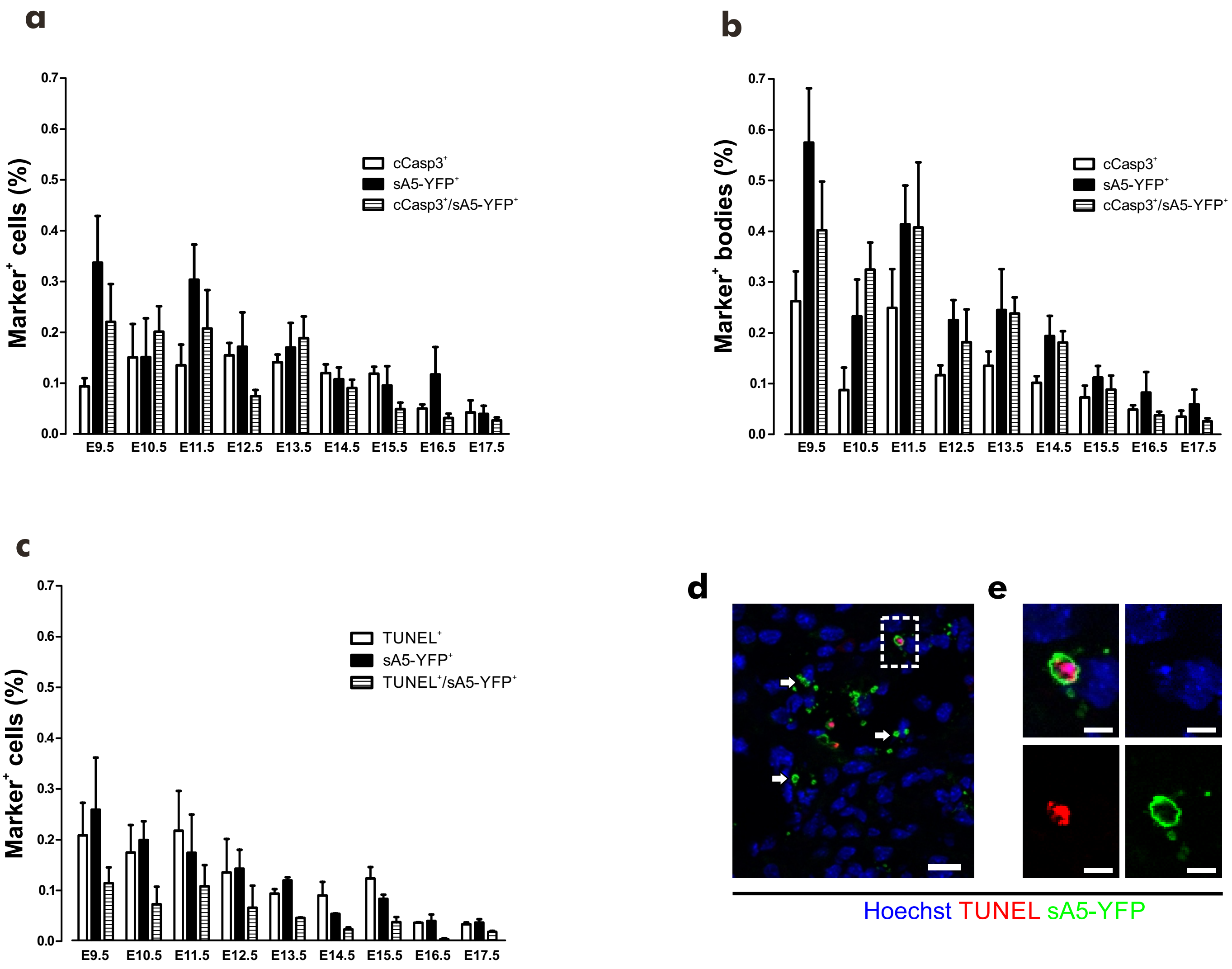

Supplement: Supplementary file 2 — Supplementary figures [file 41418_2019_426_MOESM2_ESM.pdf]
