# Supplementary material for: In vivo detection of programmed cell death during mouse heart development
Source: Cell Death Differ. 2019 Sep 30;27(4):1398–414. doi: 10.1038/s41418-019-0426-2 (PMC7205869; doi:10.1038/s41418-019-0426-2)
Supplement: Supplementary file 3 — Author contribution [file 41418_2019_426_MOESM3_ESM.pdf]

DECLARATION OF CONTRIBUTIONS TO ARTICLE

ADMC

Manuscript Number: CDD-19-0335R

Journal Name: *Cell Death & Differentiation* (the 'Journal')

Proposed Title of the Contribution:

In vivo detection of programmed cell death during mouse heart development (the 'Contribution)

Author(s):

Kristel Martínez-Lagunas, Yoshifumi Yamaguchi, Cora Becker, Caroline Geisen, Marco C DeRuiter, Masayuki Miura, Bernd K. Fleischmann and Michael Hesse (the 'Authors')

For all *CDD* articles, each person named as an author in the published version must be able to show he or she has contributed substantially to the article.

Authorship credit should be based on 1) substantial contributions to conception and design, acquisition of data, or analysis and interpretation of data; 2) drafting the article or revising it critically for important intellectual content; and 3) final approval of the version to be published. Authors should meet conditions 1, 2 and 3.

Any person who cannot be shown to have made a substantial contribution to the article cannot be listed as an author in the final version. The name of any person who is deemed to have made a minor contribution can, however, appear in the Acknowledgments section of the article.

Please complete the table below to indicate the contributions of all named authors to the manuscript.

Author Full Name:	Specification of Contribution to the Manuscript:
Kristel Martínez-Lagunas	Performed molecular biology, cell culture, imaging, and immunohistochemistry experiments and data analysis. Prepared figures and wrote the manuscript.
Yoshifumi Yamaguchi	Performed imaging of neural tube closure and data analysis. Prepared figures.
Cora Becker	Performed immunohistochemistry experiments and data analysis. Prepared Suppl Fig. 8.
Caroline Geisen	Conceived and designed the sA5-YFP expression cassette. Helped with the molecular biology experiments.
Marco C DeRuiter	Helped to design and interpret microscopic and immunostaining findings.
Masayuki Miura	Involved in imaging of neural tube closure and data analysis. Designed experiments and helped writing the manuscript.
Bernd K. Fleischmann	Designed the study and wrote the manuscript.
Michael Hesse	Was involved in the generation of sA5-YFP-mice. Conceived and designed the experiments, wrote the manuscript and prepared figures.

Please complete the table below to indicate the contributions of all named authors to the figures.

Figure 1:

KML performed the experiments.
BKF and MH designed the experiments. KML and MH prepared the figure.

Figure 2:

MH, CG and KML generated the transgenic sA5-YFP mice. KML performed the imaging. KML and MH prepared the figure.

Figure 3:

a,b) KML performed the live-imaging and did the analysis of fluorescence intensity.
c,d) YY and MM designed and performed the experiment, YY prepared the figure.

Figure 4:

KML performed the experiments.
BKF and MH designed the experiments. KML and MH prepared the figure.

Figure 5:

KML performed the experiments.
BKF and MH designed the experiments.
MCD helped to interpret microscopic and immunostaining findings. KML and MH prepared the figure.

Figure 6:

KML performed the experiments.
BKF and MH designed the experiments.
MCD helped to interpret microscopic and immunostaining findings. KML and MH prepared the figure.

Signed for and on behalf of the Author(s):	Print Name:	Date:
M. [signature]	Michael Hesse	02.09.2019
